# Plastic and elastic biomechanical properties of anterior cruciate ligament autografts

**DOI:** 10.1186/s12891-024-07262-y

**Published:** 2024-02-19

**Authors:** Mason Garcia, Kaveh Momenzadah, Mohammad Javad Shariyate, Nadim Kheir, Mohammad Khak, Juan B Villarreal, Mohammadreza Abbasian, Alexandra F Flaherty, Philip Hanna, Arun Ramappa, Nikolaos K Paschos, Ara Nazarian

**Affiliations:** 1 Musculoskeletal Translational Innovation Initiative, Beth Israel Deaconess Medical Center, Harvard Medical School, 330 Brookline Avenue, RN123, Boston, MA 02115 USA; 2 Mechanical Engineering Department, Boston University, Boston, MA USA; 3 Carl J. Shapiro Department of Orthopaedic Surgery, Beth Israel Deaconess Medical Center, Harvard Medical School, Boston, MA USA; 4 Orthopedic Surgery, Massachusetts General Hospital, Harvard Medical School, Boston, USA; 5 Department of Orthopaedic Surgery, Yerevan State Medical University, Yerevan, Armenia

**Keywords:** Anterior cruciate ligament, Anterior cruciate ligament reconstruction, Autograft, Biomechanical properties

## Abstract

**Background:**

Anterior cruciate ligament (ACL) rupture is a common orthopedic injury, occurring in roughly 68.6 per 100,000 persons annually, with the primary treatment option being ACL reconstruction. However, debate remains about the appropriate graft type for restoring the native biomechanical properties of the knee. Furthermore, plastic graft elongation may promote increased knee laxity and instability without rupture. This study aims to investigate the plastic properties of common ACL-R graft options.

**Methods:**

Patellar tendon (PT), hamstring tendon (HT), and quadriceps tendon (QT) grafts were harvested from 11 cadaveric knees (6 male and 5 female) with a mean age of 71(range 55–81). All grafts were mechanically tested under uniaxial tension until failure to determine each graft’s elastic and plastic biomechanical properties.

**Results:**

Mechanically, the QT graft was the weakest, exhibiting the lowest failure force and the lowest failure stress (QT < HT, *p* = 0.032). The PT was the stiffest of the grafts, having a significantly higher stiffness (PT > QT, *p* = 0.0002) and Young’s modulus (PT > QT, *p* = 0.001; PT > HT, *p* = 0.041). The HT graft had the highest plastic elongation at 4.01 ± 1.32 mm (HT > PT, *p* = 0.002). The post-yield behavior of the HT tendon shows increased energy storage capabilities with the highest plastic energy storage (HT > QT, *p* = 0.012) and the highest toughness (HT > QT, *p* = 0.032).

**Conclusion:**

Our study agrees with prior studies indicating that the failure load of all grafts is above the requirements for everyday activities. However, grafts may be susceptible to yielding before failure during daily activities. This may result in the eventual loss of functionality for the neo-ACL, resulting in increased knee laxity and instability.

## Background

Anterior cruciate ligament (ACL) tears are a common orthopedic injury with the peak incidence in young athletic populations. These injuries are estimated to occur in approximately 68.6 per 100,000 persons annually in the United States alone, with the definitive treatment being ACL reconstruction (ACL-R) [1]. In the past several decades, the number of ACL-R has significantly increased, with over 200,000 surgeries performed annually in the United States [1, 2]. The primary goal of ACL-R is the restoration of the native biomechanical properties of the knee joint, including anteroposterior and rotatory knee stability [3].

Despite the incidence of ACL-R, graft selection remains a topic of debate due to a need for greater consensus concerning the advantages and disadvantages of each graft option [4, 5]. For primary ACL-R, autografts have been established as superior to allografts; however, the type of autograft used remains controversial [4, 6]. The most common autograft used by orthopedic surgeons is the hamstring tendon (HT), followed by the patellar tendon (PT) and the quadriceps tendon (QT) [7]. While all three autografts have demonstrated similar clinical effectiveness in restoring the knee’s structural and biomechanical integrity [4], debate arises regarding each graft’s specific donor site comorbidities.

HT autografts are currently the most commonly utilized grafts comprising semitendinosus and gracilis tendons [8], offering a lower donor site morbidity than PT grafts [4, 9]. However, the harvesting procedure is more technically challenging, which increases the risk of graft truncation and, more importantly, increases variability in graft length and size, altering the graft’s biomechanical properties [9]. As a result, the inability to predetermine graft size is the primary concern for this option. There is concern over increased post-operative laxity compared to PT and QT grafts [9]. Additionally, residual weakness of the hamstrings is often reported as a potential risk [10].

PT grafts were the first autograft used for ACL reconstructions, historically represented as the “gold standard” in ACL-R [4]. These grafts comprise a bone plug from the tibia, a portion of the patellar tendon’s central third, and a patella bone plug [4]. The advantages of this autograft include bone-to-bone fixation on both ends of the graft, promoting bone-to-bone healing, which is mechanically stronger than soft tissue-to-bone healing [4]. Furthermore, there is a low level of technical difficulty associated with graft harvesting, which helps with predetermined graft sizes [2, 4]. However, the ease of harvesting this graft comes at the expense of anterior knee pain and an increased risk of patellar fracture [2, 4, 11].

QT grafts have received increasing interest as a reliable alternative to HT and PT grafts [12, 13]. QT grafts can comprise a bone plug from the patella and tendon or the free tendon itself, with both options showing good to excellent clinical outcomes in the 2-year follow-up period [12]. Furthermore, this graft has a lower incidence of anterior knee pain and a larger cross-sectional area, which has been shown to correlate with increased biomechanical properties [2, 4, 11]. However, the major disadvantage of harvesting the QT tendon is the risk of extensor mechanism weakness and the associated stiffness [4].

In comparing autografts, the advantages and disadvantages mentioned above are important; however, one of the key drivers of the ongoing debate is the strength of each graft, which has been shown to correlate with the cross-sectional area of the graft, graft volume, and mechanical tissue properties of the graft [4, 9]. Previous literature shows significant variability in reported biomechanical tensile strength and stiffness values between autografts. While similar trends have been reported on the relative strength of different graft options, no studies have reported on the plastic properties of these graft types, which could help assess post-operative instability due to unrecoverable (plastic) graft elongation. Specifically, it is important to understand what loads cause the grafts to yield and cause unrecoverable elongation prior to failure. Plastic deformity in the graft may lead to a non-functional neo-ACL despite the absence of any discontinuity shown on MR imaging. Thus, a better understanding of the plastic mechanical properties of ACL grafts could help assess post-operative instability due to unrecoverable (plastic) graft elongation. Therefore, we aim to analyze these biomechanical parameters to consider the plastic strain of each graft as a marker to diagnose post-operative instability before the rupture of a neo-ACL. *We hypothesize that all graft options are mechanically stiff enough to mimic native ACL function; however, the permanent elongation of the HT graft will be the highest.*

## Methods

### Graft harvesting

The grafts were obtained from the anatomy laboratory at our institution. Eleven fresh frozen right cadaveric knees (6 male and 5 female) with a mean age of 71 (range 55 to 81) were used. A surgical procedure involving a skin incision along the midline was conducted. Upon dissecting the sartorius aponeurosis, the gracilis and semitendinosus muscles were seen in the inferior region of the incision. The tendons were separated from their muscle bodies using an open tendon stripper and severed from their tibial attachment at the periosteum. The patellar tendon was obtained by a conventional technique, including extracting tibial and patellar bone blocks. The patellar and quadriceps tendons were harvested at a width of 10 mm and full thickness. The patellar tendon was extracted with 1 × 1 × 2 cm bone blocks on both ends. The quadriceps tendon was procured with a 1 × 1 × 2 cm bone block at the patellar end. The cross-sectional areas of the PT, QT, and quadrupled HT were measured using a micrometer. All grafts’ length was approximately 5.5 cm.

The grafts were thawed overnight and left to equilibrate at ambient temperature (21 °C) for a minimum of 12 h before testing. Otherwise, they were stored at – 4 °C. The aforementioned processing method has not changed tendon mechanical characteristics [14, 15].

### Mechanical testing

**Fig. 1:**
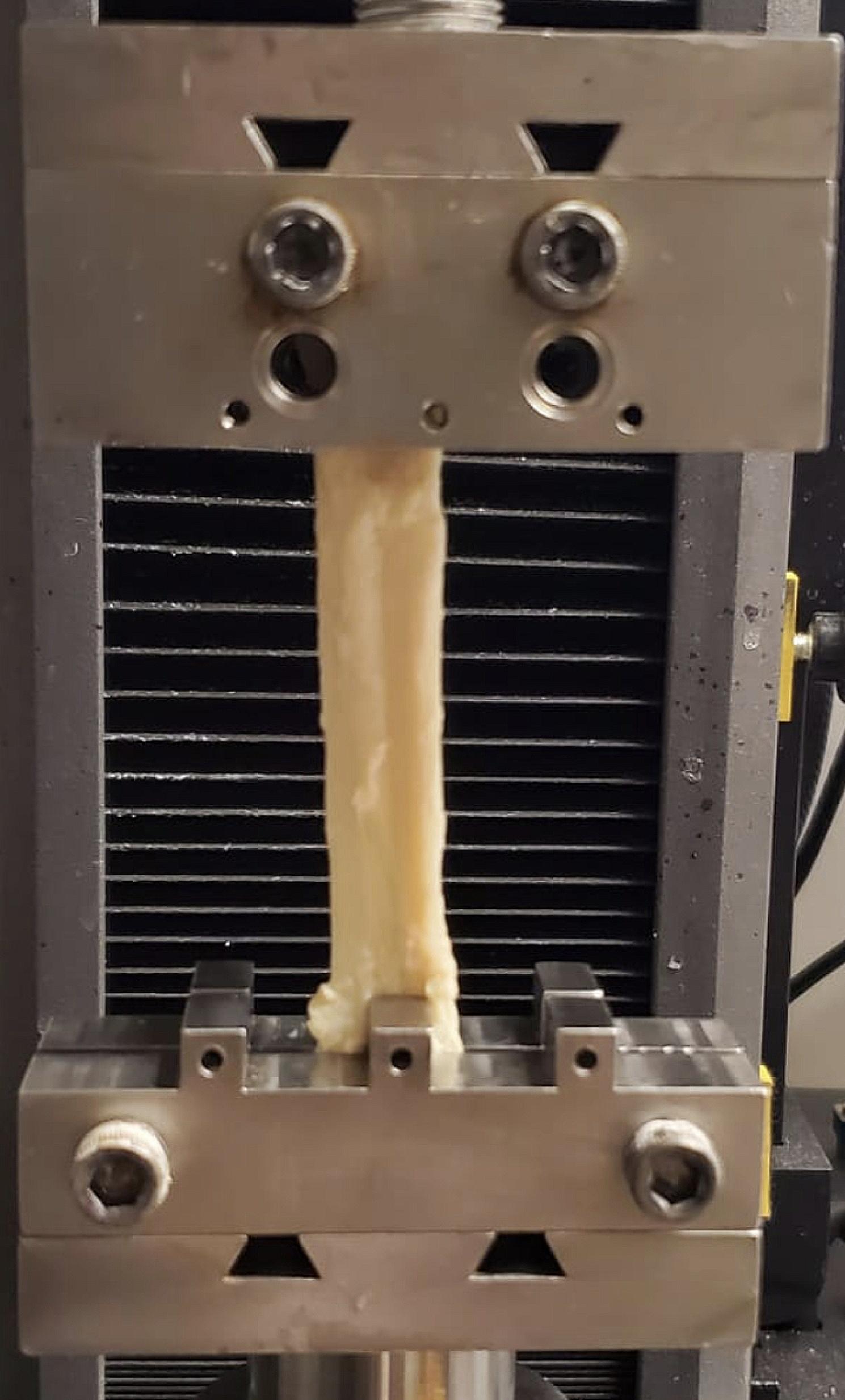
A representative setup for mechanical testing

All grafts were gripped at both ends using two serrated jaw clamps, recommended for tendon allografts [16, 17]. The PT grafts were securely held at both ends, while the QT graft was anchored at a single end using bone blocks. The grafts were then appropriately tightened to prevent slippage from the clamps and maintain the bone block’s integrity. Since the four-strand HT grafts did not have bone blocks, they were sandwiched between a nylon belt and clamped in the serrated jaws. This was done to avoid clamp slippage or crushing of the graft to alleviate the effects of premature failure. This method was verified in a pilot study to ensure tendon failure occurred within the mid-substance of the tendon and did not come from failure within the clamp. Before mechanical testing, the grip-to-grip length was recorded using a digital caliper to determine the working length of the specimen to calculate intrinsic mechanical properties. The samples were tested using a load frame (Instron 5944, Instron, Canton, MA, USA) (Fig. 1), first preloaded to 10 N to remove any slack in the system and cycled ten times from 50 to 250 N at a rate of 1 mm/s to remove any viscoelastic effects during testing [18, 19]. The grafts were loaded to failure at 100 N/s.

### Data analysis

**Fig. 2:**
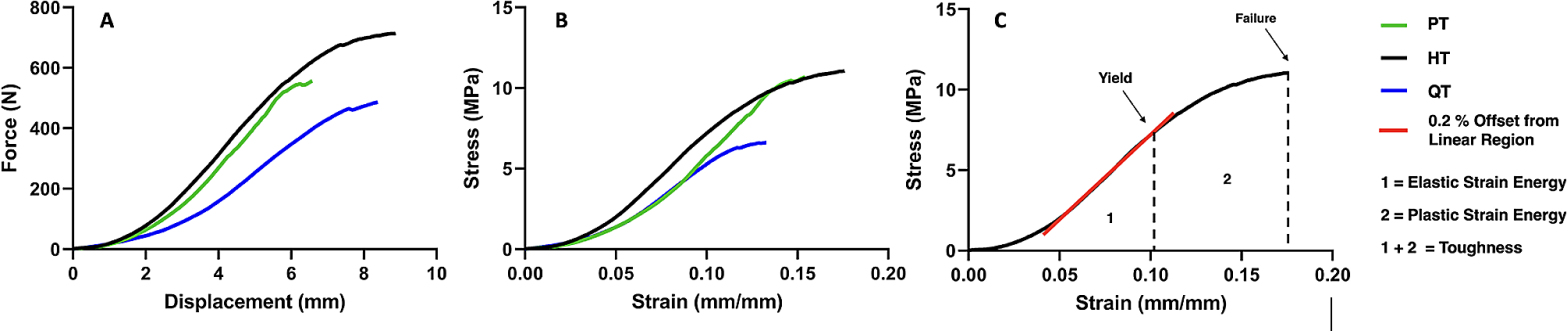
Representative plots of (**A**) the acquired load displacement, (**B**) calculated stress-strain curves used to determine the mechanical properties of each graft and (**C**) an example of calculations for mechanical properties

The load–displacement data (Fig. 2A) were analyzed using a custom-built in-house MATLAB (MathWorks, Natick, MA) to determine stiffness (N/mm), failure load (N), and displacement (mm), and subsequently converted to strength-strain curves (Fig. 2B), where strength was defined as $\sigma = \frac{F}{W*t}$ (w = width, t = thickness), and the strain was defined as $\epsilon = \frac{{\Delta }L}{L}$ (L = length), for all data points. The output consisted of the elastic modulus (MPa), yield strength (N), yield strain (mm/mm), failure strength (MPa), and failure strain (mm/mm), where the yield point was determined at 0.2% strength deviation from a linear region fit **(**Fig. 2C). The yield load was determined by multiplying the yield stress by the cross-sectional area of the graft. The plastic strain was calculated as ${\epsilon }_{Failure}- {\epsilon }_{Yield }$. To determine the energy storage properties for both the elastic and plastic regions of the grafts, the area under the curve of the strength-strain curve was calculated. The elastic energy density (J/mm^3^) was the area from the start to the yield point, the plastic energy density was from the yield point to failure, and the total energy density (toughness, J/mm^3^) was the total area under the curve until failure (Fig. 2C).

### Statistical analysis

The Shapiro-Wilk test was used to assess data distribution normality. Normal distribution was reported for all mechanical properties except yield force. For normally distributed data, one-way analysis of variance (ANOVA) was used, followed by the Tukey *post-hoc* analysis for multiple comparisons between graft options. Friedman’s ANOVA was used for non-non-normally distributed data, followed by Dunn’s *post-hoc* analysis for multiple comparisons between graft options. Statistical analysis was performed using GraphPad Prism (version 9.3.1 for Windows, GraphPad Software, San Diego, CA, USA). Two-tailed p values < 0.05 were considered significant.

## Results

The HT graft was able to withstand the most force, having the highest failure force at 828.62 ± 257.89 N, significantly stronger than the PT (*p* = 0.032) grafts. Mechanically, the QT graft was the weakest, exhibiting the lowest failure force and failure stress (QT < PT, *p* = 0.036; QT < HT, *p* = 0.015). The PT was the stiffest of the grafts, having a significantly higher stiffness (PT > QT, *p* = 0.0002) and Young’s modulus (PT > QT, *p* = 0.001; PT > HT, *p* = 0.041). Interestingly, no significant differences were observed for strain-based mechanical properties (yield, failure, and plastic strains), showing that all grafts experience similar deformation patterns. However, it should be noted that the HT graft had the highest elongation after yield at 4.01 ± 1.32 mm (HT > PT, *p* = 0.002). Overall, the QT grafts were mechanically weaker than the PT and HT, having the lowest stiffness, elastic modulus, failure strength, and energy storage capabilities. While the yield force was similar for all grafts, the post-yield behavior of the HT tendon shows greater plastic energy storage capabilities (HT > QT, *p* = 0.012) and the highest toughness (HT > QT, *p* = 0.032). Table 1 highlights all mechanical properties, with Table 2 providing details on statistical results.

**Table 1:** Mechanical properties measured for each autograft type (QT, BTB, HT). For all normally distributed data mean and standard deviation are provided. For non-normally distributed data (yield force), median and interquartile range are provided

Mechanical property	Quadriceps tendon (QT)	Patellar tendon (PT)	Hamstring tendon (HT)
Cross Sectional Area (mm^2^)	76.88 ± 13.42	51.77 ± 8.11	72.20 ± 11.80
Failure Force (N)	579.32 ± 155.43	593.03 ± 177.41	828.62 ± 257.89
Stiffness (N/mm)	116.35 ± 51.11	221.24 ± 27.34	192.27 ± 53.46
Youngs Modulus (MPa)	110.81 ± 25.29	207.13 ± 51.02	142.90 ± 40.25
Yield Force (N)	263.90 (76.80)	262.10 (97.70)	325.60 (93.80)
Yield Stress (MPa)	3.54 ± 1.08	6.54 ± 2.93	5.14 ± 1.76
Yield Strain (mm/mm)	0.116 ± 0.042	0.159 ± 0.059	0.128 ± 0.042
Failure Stress (MPa)	7.47 ± 2.27	11.45 ± 3.40	11.77 ± 4.20
Failure Strain (mm/mm)	0.169 ± 0.054	0.205 ± 0.074	0.207 ± 0.041
Plastic Strain (mm/mm)	0.053 ± 0.020	0.046 ± 0.029	0.078 ± 0.032
Elongation After Yield (mm)	3.60 ± 1.73	2.01 ± 0.98	4.01 ± 1.32
Elastic Energy Density (J/m^3^)	0.26 ± 0.19	0.33 ± 0.19	0.42 ± 0.23
Toughness (J/m^3^)	0.42 ± 0.26	0.63 ± 0.33	0.90 ± 0.40
Plastic Energy Density (J/m^3^)	0.16 ± 0.09	0.30 ± 0.30	0.48 ± 0.33

**Table 2:** Statistical results between graft types

Mechanical property	QT vs PT	QT vs HT	PT vs HT
Cross Sectional Area (mm^2^)	0.002	0.418	0.001
Failure Force (N)	0.986	0.032	0.110
Stiffness (N/mm)	0.0002	0.079	0.332
Youngs Modulus (MPa)	0 001	0.064	0.041
Yield Force (N)	> 0.999	0.6025	> 0.999
Yield Stress (MPa)	0.036	0.018	0.494
Yield Strain (mm/mm)	0.113	0.706	0.365
Failure Stress (MPa)	0.064	0.015	0.984
Failure Strain (mm/mm)	0.431	0.145	0.998
Plastic Strain (mm/mm)	0.852	0.150	0.056
Elongation After Yield (mm)	0.282	0.635	0.002
Elastic Energy Density (J/m^3^)	0.637	0.012	0.625
Toughness (J/m^3^)	0.986	0.032	0.101
Plastic Energy Density (J/m^3^)	0.494	0.043	0.362

## Discussion

The outcome of an ACL reconstruction procedure is influenced by many key elements, including the positioning of the tunnels, the type of the graft used, preconditioning of the graft, fixation of the graft to the femur and tibia, and the effectiveness of the rehabilitation process [20]. Extensive research has been conducted to investigate the biomechanical characteristics of different graft options to identify the most suitable graft source that closely replicates the biomechanics of the native ACL [5, 21]. However, there is a lack of agreement about the graft source that exhibits the highest level of biomechanical superiority compared to other sources. Previous research has mostly examined failure load and stiffness and reported failure loads equal to or more than those of the native ACL [22]. Before graft failure occurs, the plastic deformity of the neo-ACL may render the graft nonfunctional despite the absence of a tear. To our knowledge, no study has evaluated the plastic behavior of ACL autografts. Yet, understanding the plastic behavior of common graft options may provide insight into the likelihood of graft elongation or failure that causes knee instability, particularly in a scenario where a patient presenting with recent knee trauma exhibits clinical signs of knee instability and an intact neo-ACL revealed by magnetic resonance imaging (MRI). Furthermore, in post-operative rehabilitation, knowing the threshold for plastic deformity may help to improve clinical outcomes by avoiding exercises that may cause graft elongation.

The forces exerted on the ACL in its natural position are 169 N when walking, less than 100 N during stair ascending, and 445 N during stair descending [23–25]. Common physical therapy exercises, such as a single leg squat or a double-foot drop landing stepping off a 60-cm platform, resulted in peak tensile forces of 124 and 253 N, respectively [26]. Previous studies on older ACL cadaveric specimens have reported a failure load of 496 ± 85 N with a stiffness of 124 ± 16 N/mm, most comparable to our obtained data [27]. Based on this finding, it has been postulated that graft ultimate failure loads should surpass this threshold to be considered a viable substitute for the ACL. Although the failure load of grafts in our study exceeded this threshold, our investigation demonstrated that all tested grafts’ yield forces were lower than the forces put on the ACL during activities such as stair descending and perhaps contact sports, making the neo-ACL susceptible to plastic deformity even during daily activity. When observing the elongation after yield, it becomes apparent that there is a significant rise in elongation in the HT group compared to the PT group. This elongation discrepancy may contribute to the worse clinical outcomes regarding failure rate [28]. Collagen breakdown due to repetitive fatigue damage over time can increase the risk of structural failure [29]. While the ACL grafts tested experienced higher failure forces than forces seen *in-vivo*, the yield force is below this threshold; thus, one would expect that repetitive loading would result in micro-damage that ultimately fails.

On the other hand, clinical failure of ACL reconstruction is often characterized as the presence of a side-to-side discrepancy of 5 mm or more during laxity testing [30]. The average range of QT and HT elongation recorded in our investigation included this threshold, indicating that these two grafts may experience elongation, leading to clinical failure without any tendon rupture under cyclic loading. Despite our findings, the degree of graft elongation, primarily determined by the intrinsic features of the graft type, may have limited importance in attaining a satisfactory outcome in ACL restoration. Other crucial elements in determining the overall outcome of the treatment include the choice of graft fixation, the precision of the fixation procedure, and the integrity of the bone where the graft is fixed. Taking this information into account facilitates a more accurate understanding of the practical implementation of the data derived from our research.

Like tendons, ligaments have energy storage capabilities to help enable rapid, efficient force transfer and recoil to return energy to the system to promote movement [31]. Thus, considering energy storage is important when evaluating graft options, as differences in energy storage may play a role in altered knee mechanics. We observed that the HT graft has the highest plastic energy storage, meaning there is a buildup of energy within the graft during motion. While this is important to resisting rupture, high energy storage after plastic deformation could lead to altered knee mechanics. Graft elongation plus the buildup of energy during movement may lead to decreased recoil times, which could be one explanation as to why non-ruptured grafts show instability.

Previously, literature has largely examined the failure properties of common ACL grafts, mostly showing that the HT is mechanically the strongest with the highest failure load, and the PT is the stiffest of the grafts [22, 32–34]. Although reported mechanical values between studies vary significantly, this may be attributed to testing setup, graft preparation, or specimen age. Nonetheless, our studies agree with the trends noted above. It should be mentioned that the mechanical properties are on the lower end of reported data. Yet, no studies have evaluated the plastic properties of ACL grafts, which may provide insight into graft failure and knee instability before rupture, along with the energy storage capabilities. However, more work is needed to assess the effects of plastic deformation and energy storage of the ACL *in-vivo* to fully understand if these grafts may fully mimic the native mechanical properties of the ACL. Furthermore, there has been an increased interest in biomimetic ACL graft options [35–38]. Providing a complete understanding of common graft options’ elastic, plastic, and energy storage capabilities may provide insight into mimicking the mechanical properties of these tissues when developing engineered tissue for ACL-R.

The hypothesis regarding plastic deformity of grafts posits that the extension of the neo-ACL beyond its yield forces may be a contributing factor to the manifestation of knee instability in patients who have undergone ACL-R, specifically within the HT graft, as it elongates roughly 4 mm after yield, which may be clinically significant. Although we have shown that QT and PT have the potential to exhibit plastic elongation when subjected to external forces during routine activities, the elongation may not be enough to be clinically significant.

### Limitations

A primary limitation of this study is the age of the cadaveric specimens used to examine the biomechanical properties of graft type, as degeneration is typically seen with age, reducing the strength of the soft tissue [39]. However, our findings are within the reported range of mechanical properties of ACL grafts, according to a recent systematic review [22]. While they may be on the lower end of previously reported mechanical results, we see similar trends, where the PT is the stiffest graft option, and HT has the highest load to failure, consistent with other previous studies [32–34].

## Conclusion

Our study findings align with prior research, indicating that all grafts’ failure load and stiffness are above the requirements for everyday activities. However, the yield load is of concern, which falls below the required threshold for high intensity daily activities. Thus, repetitive loading above this threshold over time may result in permanent (plastic) graft elongation. Irrecoverable elongation of the graft could result in eventual instability and decreased functionality for the neo-ACL dispute, with no tear present. Additional investigation is required to assess the impact of plastic deformation on the functional performance of a neo-ACL in-vivo.

## Data Availability

The datasets used and/or analyzed during the current study is available from the corresponding author on reasonable request.

## Abbreviations

PT: Patellar tendon
HT: Hamstring tendon
QT: Quadriceps tendon
ACL: Anterior cruciate ligament
ACL-R: Anterior cruciate ligament reconstruction
